# The Role of Sphingosine-1-Phosphate Signaling in Cerebral Ischemia/Reperfusion Injury and Alzheimer’s Disease Pathology

**DOI:** 10.3390/ijms27125200

**Published:** 2026-06-09

**Authors:** Kinga Czubowicz, Joanna Agata Motyl, Agnieszka Wencel, Robert Piotr Strosznajder

**Affiliations:** 1 Laboratory of Preclinical Research and Environmental Agents, Mossakowski Medical Research Institute, Polish Academy of Sciences, 5 Pawinskiego St., 02-106 Warsaw, Poland; kczubowicz@imdik.pan.pl; 2 Laboratory of Tissue Engineering, Department of Hybrid and Analytical Microbiosystems, Nalecz Institute of Biocybernetics and Biomedical Engineering, Polish Academy of Sciences, Ks. Trojdena 4 St., 02-109 Warsaw, Poland; jmotyl@ibib.waw.pl (J.A.M.); awencel@ibib.waw.pl (A.W.)

**Keywords:** bioactive sphingolipids, sphingosine-1-phosphate receptors modulators, brain ischemia, inflammation, Alzheimer’s disease, neurodegeneration, neuroprotection

## Abstract

Sphingosine-1-phosphate (S1P) is a pleiotropic bioactive sphingolipid that regulates key cellular processes, like proliferation, apoptosis, inflammation, and vascular homeostasis. S1P acts as a signaling molecule both inside and outside cells by interacting with five G-protein-coupled S1P receptors (S1PR1–S1PR5). Accumulating evidence indicates that dysregulation of S1P signaling is implicated in the pathophysiology of cerebral ischemia/reperfusion (I/R) injury and Alzheimer’s disease (AD). In I/R injury, S1P signaling regulates vascular permeability, immune cell infiltration, and neuronal survival and death. In AD, alterations in S1P metabolism are associated with β-amyloid deposition, tau hyperphosphorylation, synaptic dysfunction, and sustained neuroinflammation. S1P receptor (S1PR) modulators represent promising therapeutic agents in both preclinical and clinical studies. Fingolimod was the first oral disease-modifying therapy approved for the treatment of multiple sclerosis and, at the same time, the first S1PR modulator introduced into clinical practice. New selective S1PR-targeting agents, including siponimod and ozanimod (S1PR1 and S1PR5), as well as the S1PR1-selective agent ponesimod, have also been approved for clinical use. In addition to their immunomodulatory properties, S1PR modulators have direct effects in the central nervous system, facilitating the maintenance of blood–brain barrier integrity, reducing microglial activation, and enhancing neuronal survival pathways. Building on this knowledge, we discuss the role of S1P signaling, highlighting recent advances in S1PR modulators as promising therapeutic agents for cerebral I/R injury and AD.

## 1. Introduction

Sphingosine-1-phosphate (S1P), a bioactive sphingolipid metabolite, is found in many biological fluids, including blood and lymph, as well as various cell types, such as red blood cells, neutrophils, platelets [1,2]. In physiological conditions, S1P contributes to cell proliferation, prevents apoptosis, and regulates cell adhesion mechanisms [3]. Moreover, it has been implicated as a potential therapeutic target for numerous diseases, including cancer, atherosclerosis, diabetes, neurodegenerative diseases, and autoimmune disorders [4,5,6,7]. Outside the cell, S1P executes its biological functions in an autocrine or paracrine manner, predominantly by interacting with S1P receptors (S1PRs), a distinct group of G protein-coupled receptors (GPCRs). These receptors are classified into five subtypes (S1PR1-S1PR5) and exhibit different or overlapping expression patterns. They are ubiquitously expressed on the membranes of various cells, including lymphocytes, cardiac myocytes, endothelial cells, glia, and neurons [8,9]. The intracellular concentration of S1P is precisely controlled by the equilibrium between its production through sphingosine kinases (Sphk1/2) and its irreversible or reversible degradation by S1P phosphatases (SGPPs) and S1P lyase (SGPL1) [10]. Mammalian cells contain two Sphks: Sphk1 is present in the cytoplasm, whereas Sphk2 is present in several organelles, including the nucleus. Sphk1 activity is mainly linked to cell survival, whereas Sphk2 exhibits dual, contrasting roles. On one hand, Sphk2 supports physiological regulation, affecting mitochondrial function and gene expression through HDAC1 inhibition. On the other hand, Sphk2 has also been implicated in the suppression of cell growth and the promotion of apoptosis [11,12,13,14].

Extensive research has demonstrated the involvement of S1P in both ischemic brain injury and Alzheimer’s disease (AD) [7,15,16,17]. Epidemiological data further indicate that cerebral ischemia increases the risk of developing dementia. These studies have established that AD and cerebral ischemia share several overlapping pathological mechanisms, with neuroinflammation being a central molecular event in both conditions [18,19,20,21,22,23]. Following cerebral ischemia and reperfusion injury, resident microglia and astrocytes are rapidly activated, resulting in the release of pro-inflammatory cytokines, chemokines, reactive oxygen species (ROS), nitric oxide, and matrix metalloproteinases. Although the initial inflammatory response may support tissue repair and facilitate the removal of cellular debris, sustained activation of inflammatory pathways can be detrimental, promoting neuronal loss and exacerbating secondary brain damage [24,25,26]. A comparable process occurs in Alzheimer’s disease, where prolonged activation of microglia and astrocytes contributes to progressive neurodegeneration, synaptic dysfunction, and cognitive decline. Furthermore, the accumulation of amyloid-β and hyperphosphorylated tau proteins amplifies inflammatory signaling through pathways such as NF-κB and NLR Family Pyrin Domain-Containing 3 (NLRP3) inflammasome activation, leading to increased release of pro-inflammatory mediators [27,28,29]. Disruption of the blood–brain barrier (BBB) is another key feature common to both cerebral ischemia and Alzheimer’s disease. After ischemic injury, the BBB rapidly deteriorates, facilitating edema formation, infiltration of peripheral immune cells, oxidative stress, and subsequent neuronal damage. Emerging evidence suggests that BBB dysfunction is not solely a consequence of AD but may also represent an early event that contributes to disease onset and progression by promoting neuroinflammation, vascular impairment, and impaired clearance of misfolded proteins from the brain [30,31]. Established risk factors for AD, such as aging, type 2 diabetes mellitus, the ApoE genotype, and elevated serum cholesterol levels, are frequently linked to vascular dysfunction, which in turn contributes to the development of cerebral ischemia [23].

Recent evidence suggests that AD is not exclusively a brain disorder but also involves pathological changes within the gut–liver–brain axis. In addition to the classical hallmarks of AD, such as Aβ accumulation and tau pathology, there is growing interest in peripheral mechanisms that may contribute to neurodegeneration. Impaired Aβ clearance is recognized as a significant factor in disease progression. As the liver is the primary site of peripheral Aβ metabolism, hepatic dysfunction may reduce Aβ clearance and facilitate its accumulation in the brain. Disturbances in gut barrier integrity and liver function often result in chronic low-grade inflammation, increasing circulating pro-inflammatory mediators such as IL-6 and TNF-α. These factors can compromise BBB integrity, promote neuroinflammation, and accelerate neurodegenerative changes. Altered bile acid metabolism, observed in AD patients, may further contribute to these processes [32,33,34].

Previous and current investigations of the S1PR signaling pathway have led to the approval of drugs and the identification of potential new targets for further therapeutic intervention. Fingolimod (FTY720; Gilenya^®^) represented the first S1PR modulator approved for the treatment of patients with relapsing forms of multiple sclerosis (RMS) [35]. The development of second-generation S1PR modulators, such as ozanimod, ponesimod, and siponimod, followed. These modulators exhibit diverse mechanisms of action by working as agonists with functional antagonism or as classical agonists [36,37,38,39,40]. In addition, some modulators function as prodrugs and require phosphorylation to become biologically active; for example, FTY720 is phosphorylated mainly by Sphk2. In contrast, second-generation S1PR modulators such as siponimod include chemical groups that structurally resemble the terminal phosphate moiety of S1P and thus do not require phosphorylation to become active [39].

## 2. The Sphingosine-1-Phosphate Axis in Neuroinflammation and Blood–Brain Barrier Dysfunction Following Cerebral Ischemia–Reperfusion Injury

Increasing evidence identifies that S1P signaling plays a significant role in regulating both neuroinflammatory responses and BBB integrity after ischemic stroke. Among the enzymes involved in S1P metabolism, Sphk1/2 appear to exert distinct functions during ischemic injury. Several studies have indicated that Sphk1 plays a crucial role in mediating post-stroke neuroinflammation [16,41]. Both mRNA/protein levels of Sphk1 were persistently elevated in the cortical penumbra following middle cerebral artery occlusion (MCAO), and this increase was maintained for up to 96 h after reperfusion, whereas Sphk2 mRNA expression remained unchanged. Furthermore, both Sphk1 pharmacological inhibition and its genetic knockdown significantly improved ischemic outcomes, including reduced infarct volume, improved neurological function, and decreased production of pro-inflammatory mediators such as IL-1β, iNOS, IL-6, and TNF-α [42]. Enhanced Sphk1 expression has been primarily associated with activated microglia. Moreover, Sphk1 suppression decreased TNF receptor-associated factor 2 (TRAF2) and NFκB expression/protein levels in a rat I/R injury model. Experimental data demonstrated that Sphk1 modulates IL-17A production through the TRAF2/NFκB pathway [43]. Furthermore, S1P administered to oxygen-glucose deprivation/reoxygenation (OGDR)-treated microglia enhanced IL-17A secretion compared to OGDR alone and further increased neuronal apoptosis [44].

An additional mechanism underlying the activity of Sphk1 was described by Ader et al. (2008), who showed that under hypoxic conditions, Sphk1 modulates HIF-1α (hypoxia-inducible factor-1) protein levels through phosphorylation of Akt/GSK3β (Protein Kinase B/Glycogen Synthase Kinase 3 beta) kinases and prevents its proteasomal degradation [45].

**Figure 1:**
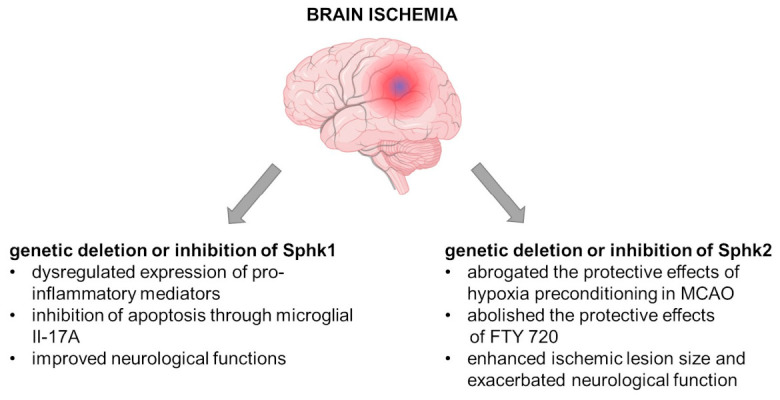
The role of sphingosine kinases 1 and 2 (Sphk1, Sphk2) in cerebral ischemia. The figure illustrates that experimental genetic deletion or inhibition of sphingosine kinases causes different effects in cerebral ischemia–reperfusion injury.

Unlike Sphk1, many studies have reported beneficial roles for Sphk2 in cerebral ischemia (see Figure 1 for a summary of the roles of Sphk1 and Sphk2).

**Figure 2:**
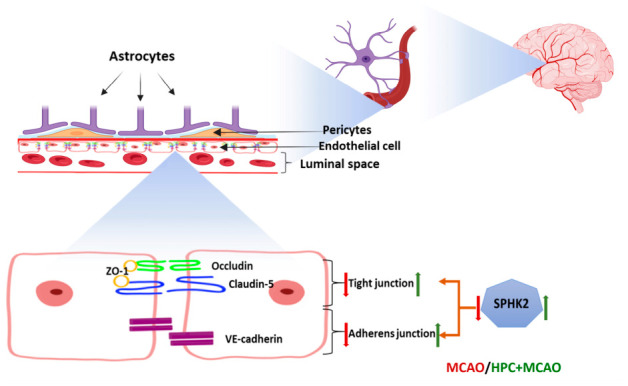
The influence of sphingosine kinase 2 on blood–brain barrier maintenance in cerebral ischemia. The figure illustrates the schematic drawing of the blood–brain barrier (BBB) showing the endothelium, basement membrane, pericytes, and astrocytes. Furthermore, the figure shows the simplified representation of protein interactions associated with tight junctions at the BBB. Claudin, occludin, and junction adhesion molecule – VE-cadherin are the transmembrane proteins, and zonula occludens-1 (ZO-1) is the cytoplasmic protein. The sphingosine kinase 2 (Sphk2) is an important mediator for regulating the adherens junction protein VE-cadherin, and the tight junction proteins: claudin-5, occludin, and ZO-1 in hypoxia preconditioning (HPC)-induced neuroprotection after cerebral ischemia. Arrows terminating with ↑ = increased, arrows terminating with ↓ = decreased.

Sphk2 is suggested to be important for preconditioning-induced neuroprotection in cerebral ischemia [46,47,48]. Studies showed that Sphk2, but not Sphk1, was rapidly and transiently upregulated in mouse brains after hypoxic preconditioning. Hypoxia-mediated neuroprotection significantly reduces infarct volume and improves outcomes in wild-type and Sphk1^−/−^ mice. The protection is not seen in Sphk2^−/−^ mice [48]. In neurons subjected to OGDR, Sphk2 expression was increased, which was associated with the activation of autophagy and neuroprotection [49]. Overexpression of Sphk2 in primary murine cortical neurons and HT22 hippocampal cells led to elevated LC3-II protein levels and enhanced autophagic flux. It has been suggested that Sphk2, via its BH3 domain, may interact with Bcl-2, disrupting the Beclin-1/Bcl-2 complex and initiating autophagy [49]. Allicin, a natural compound derived from garlic, has been reported to increase Sphk2 expression, which in turn reduces both MCAO-induced brain injury and OGDR-induced neuronal death. These neuroprotective effects were partially abolished by the Sphk2 inhibitor ABC294640 [50], suggesting that Sphk2 activation may constitute an endogenous protective mechanism during cerebral ischemia. Accordingly, Sphk2^−/−^ mice displayed larger infarct volumes and worse neurological outcomes after MCAO [51]. Furthermore, the study reported that reduced protein levels of tight junction proteins (ZO-1, occludin) and the adherens junction protein (VE-cadherin) in ischemic wild-type mice were prevented by hypoxic preconditioning and mediated by Sphk2, since this protection was not seen in Sphk2^−/−^ mice [44] (Figure 2).

The S1PR signaling pathway plays a key role in regulating vascular endothelial permeability and sustaining endothelial barrier function. Among the S1PRs, S1PR1, S1PR2, and S1PR3 are expressed by brain endothelial cells and modulate vascular permeability and cellular activation [52]. S1PRs differentially regulate blood–brain barrier (BBB) permeability. S1PR1 activation drives Rac-dependent signaling, elevates adhesion protein expression and assembly, reinforces intercellular junctions, and stabilizes endothelial barrier integrity. Conversely, S1PR2 activation increases BBB permeability by stimulating the Rho pathway. Rho signaling induces cytoskeletal contraction, stress fiber formation, and junctional disruption, leading to elevated endothelial permeability and barrier dysfunction [53,54]. It has also been reported that S1PR5 may play a role in sustaining BBB integrity and influencing endothelial inflammatory responses [55]. Regulation of S1PR5, both at the mRNA and protein levels, in oligodendrocytes and brain endothelial cells within white matter tracts has been shown to support myelin regeneration and prevent synaptic dysfunction, ultimately reducing neuronal damage. Knockdown of S1PR5 decreased the expression of tight junction- and adherens junction-associated proteins, including claudin-5 and VE-cadherin, which are essential for BBB integrity, and enhanced the transendothelial migration of monocytes. This was accompanied by increased expression of proinflammatory and leukocyte adhesion molecules such as intercellular adhesion molecule (ICAM-1) and vascular cell adhesion molecule (VCAM-1), a process mediated by NF-κB activation. Consequently, pharmacological modulation of S1PR5 with an agonist may appear as a promising therapeutic strategy for stroke, as activation of the S1PR5 signaling pathway reduces neurovascular inflammation and leukocyte migration [55].

Studies have demonstrated that modulation of S1PR1 using pharmacological compounds may trigger neuroprotective effects in ischemic stroke by activating anti-apoptotic signaling pathways. In experimental models of MCAO, S1PR1 modulation with FTY720 (a modulator of S1PR receptors, mainly S1PR1, but also S1PR3, S1PR4, and S1PR5) was associated with reduced infarct volume and improved neurological outcomes [56,57]. In contrast, inhibition of S1PR1 with the non-selective antagonist VPC20319 abolished the anti-apoptotic effects. Furthermore, treatment with FTY720 significantly increased the levels of phosphorylated Akt and Extracellular Signal-Regulated Kinase (ERK) proteins [56,57]. The S1PR1 receptor signaling pathway may also protect against stroke by regulating endothelial cell function. Induction of S1PR1 with SEW2871 enhanced collateral vessel formation in a common carotid artery occlusion (CCAO) model, resulting in reduced infarct size and better neurological function after subsequent MCAO [56,57]. Supporting these findings, an *in vivo* study showed that the selective S1PR1 agonist LASW1238 significantly decreased the area of cerebral infarction after middle cerebral artery ischemia/reperfusion (I/R) injury [56,57]. In contrast to the results described above in animal models of MCAO, inhibition of S1PR1 expression through pharmacological antagonists or genetic knockdown suppressed microglial proliferation, reduced microglia-mediated inflammatory responses and ICAM-1 expression, and consequently improved BBB integrity. Furthermore, genetic knockdown of S1PR1 prevented the decrease in brain-derived neurotrophic factor (BDNF) expression in the ischemic brain [56,57]. Although accumulating evidence suggests that S1PR1 signaling plays a protective role in cerebral ischemia, the precise mechanisms underlying its actions remain unclear. It should be noted that the majority of evidence supporting a neuroprotective role of S1PR1 in cerebral ischemia has been derived from studies employing pharmacological modulators rather than receptor-specific genetic approaches, making it difficult to determine whether S1PR1 activation itself is beneficial or detrimental under ischemic conditions. The interpretation of these findings is further complicated by the pharmacological characteristics of certain S1P receptor modulators. For instance, after phosphorylation, fingolimod (FTY720-P) can induce S1PR1 internalization and functional desensitization in addition to receptor activation. Consequently, its neuroprotective effects may result from a combination of receptor activation, immunomodulation, and regulation of S1PR1 signaling [58,59]. Taken together, these observations suggest that the effects of S1PR1 modulation are highly context-dependent and may vary according to several factors, including the stage and severity of ischemic injury, cell-specific receptor expression, the timing and duration of receptor modulation, and differences between transient receptor activation and prolonged receptor desensitization.

**Figure 3:**
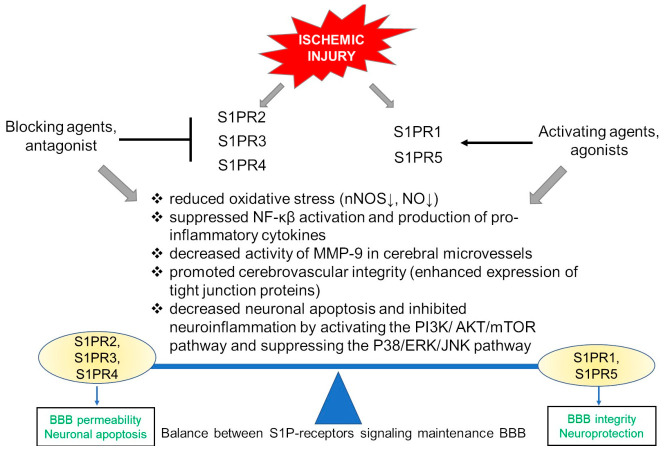
The role of the sphingosine-1-phosphate receptor signaling pathway in cerebral ischemia. The figure shows the balance of the sphingosine-1-phosphate (S1P) signaling pathway in maintaining the blood–brain barrier (BBB) in ischemia–reperfusion injury. Activators of S1PR1 and S1PR5 receptors exert the protective effects through the mechanisms presented in the figure. Furthermore, in contrast to S1PR1 and S1PR5, the blocking agents of S1PR2, S1PR3, and S1PR4 receptors are widely studied as promising protectants in ischemic injury.

The S1PR2 and S1PR3 contribute to post-ischemic brain injury through mechanisms involving microglial activation, morphological transformation into an amoeboid form, and increased proliferation within the ischemic penumbra [16,60]. Kim et al. (2015) demonstrated that S1PR2 increases cerebrovascular permeability after middle cerebral artery occlusion (MCAO) [61] by activating MMP-9 in endothelial cells. Both genetic deletion and pharmacological inhibition of S1PR2 with JTE013 preserved cerebrovascular integrity, reduced neuronal death, and improved neurological recovery. Inhibition of S1PR2 also attenuated cerebral edema and spontaneous hemorrhagic transformation in experimental stroke models. The induction of MMP-9 by S1PR2 involves the Rho–ROCK–NF-κB and stress-activated protein kinase signaling pathways [54]. Fan et al. (2022) further demonstrated that S1PR3 is involved in the regulation of BBB dysfunction [62,63]. The authors observed enhanced S1PR3 mRNA levels after MCAO in mice. Administration of CAY10444, a selective S1PR3 inhibitor, significantly attenuated brain edema and improved neurological deficits. Furthermore, CAY10444 increased the expression of tight junction proteins, including zonula occludens-1 (ZO-1) and occludin, whose levels were reduced after MCAO. Finally, the study showed that S1PR3 regulates the MAPK and PI3K–Akt signaling pathways. Inhibition of S1PR3 also reduced nNOS expression and nitric oxide (NO) production, indicating a role in regulating oxidative stress responses following cerebral I/R [62,63]. It has been described that mRNA levels of multiple S1PRs and, consistently, the protein levels were up-regulated in the mouse brain following MCAO [64]. The expression of S1PR1, S1PR3, and S1PR5 was transiently induced in the *peri*-infarct lesion with a peak observed at 1 day after MCAO. The expression of S1PR2 was induced in the inner layer of vessels in the ischemic core at the acute phase after MCAO [65]. Mazzantini et al. (2025) found enhanced mRNA levels of S1PR2 and S1PR3 in organotypic hippocampal slices after OGD treatment [66]. Unlike previous literature, no changes in the mRNA expression levels of Sphk1 and Sphk2 were observed. The selective antagonists JTE-013 and CAY10444, targeting S1PR2 and S1PR3, respectively, reduced CA1 damage caused by OGD. In this study, OGD increased TREM2 (triggering receptor expressed on myeloid cells 2) expression [66]. It was suggested that S1P could regulate microglial phagocytosis [67] and bind to TREM2, thereby improving neuroprotection in ischemic and hemorrhagic stroke [68]. In this study, the increase in TREM2 was prevented by treatment with both S1PR2 and S1PR3 antagonists. Additionally, Mu et al. (2026) demonstrated that both pharmacological inhibition and genetic knockdown of S1PR3 in cerebrovascular endothelial cells (BMECs bEnd3 cells) further increased H_2_O_2_-induced endothelial hyperpermeability, ZO-1 alteration, ROS accumulation, and reduced cell viability [69]. They also observed enhanced phosphorylation of p38, ERK, cPLA_2_, JNK, and STAT3. A subsequent study highlighted the role of the S1PR4 receptor as a novel contributor to the pathogenesis of ischemic stroke [70]. The results revealed that the functional antagonist of S1PR4 receptor, NXC736, shows protective activity against ischemic stroke by mitigating cerebral infarct development, neurological dysfunction, and neuronal cell apoptosis. This antagonist also affects the brain’s inflammatory response after stroke through the regulation of the NLRP3 inflammasome, NF-κB, and MAPKs. Pharmacological inhibition with NXC736 or genetic silencing of S1PR4 blocked BBB disruption, limited neutrophil infiltration, suppressed microglial activation and proliferation, and decreased pro-inflammatory cytokine expression [70]. In another study, Guo et al. (2025) reported that S1PR5 expression was significantly increased in the infarct penumbra following cerebral I/R injury [71]. The authors further showed that A-971432, a selective S1PR5 agonist, reduced infarct size and promoted neurological function in mice subjected to I/R injury. *In vivo*, A-971432 significantly attenuated neuronal apoptosis and inhibited neuroinflammation by stimulating the PI3K/AKT/mTOR pathway. It also suppressed p38/ERK/JNK signaling. Conversely, silencing S1PR5 exacerbated neuronal apoptosis and inflammatory responses. This led to increased cerebral infarct volume and more severe neurological impairments [71] (see Figure 3 for a summary).

## 3. The Role of Ischemic Injury in β-Amyloid and Tau Metabolism

It has been observed that ischemic brain injury may affect β-amyloid (Aβ) metabolism. The results showed that diffuse and senile amyloid plaques were observed in areas of the brain prone to ischemia, in the cerebral cortex, and in the arterial border zones after brain injury [18,19,20,21,22,23,72]. Following ischemia, soluble Aβ is transported from the circulation into the brain, contributing to amyloidosis, plaque formation, and cerebral amyloid angiopathy [19]. Changes in gene expression involved in Aβ precursor protein (APP) processing following cerebral ischemia were described with emphasis on hippocampal subregions (CA1, CA3) and the medial temporal cortex (Table 1). Downregulation of α-secretase (ADAM10) mRNA levels was observed in the CA3 region at all examined time points (2, 7, and 30 days post-ischemia). Similarly, ADAM17 expression and activity were reduced in the hippocampus under conditions of chronic cerebral hypoperfusion, indicating suppression of the non-amyloidogenic pathway. In contrast, β-site APP cleaving enzyme-1 (BACE1) exhibited a region- and time-dependent expression pattern. In the CA1 region, BACE1 mRNA levels increased at early time points (2–7 days), and then decreased at 30 days. In the CA3 region, the opposite trend was observed: decreased expression at 2 and 7 days, followed by an increase at 30 days. In the medial temporal cortex, BACE1 expression was transiently elevated at 2 days, followed by downregulation at later time points (7 and 30 days). Expression of γ-secretase components also showed dynamic modulation of gene expression. PSEN1 mRNA levels were increased in both the CA1 and CA3 regions at 2 and 7 days post-ischemia, with a reduction observed at 30 days. No significant changes were detected in the medial temporal cortex. PSEN2 expression demonstrated a more complex expression profile, with enhanced mRNA levels in the CA1 region at 2 and 7 days, followed by a decrease at 30 days. In contrast, CA3 showed reduced PSEN2 expression at early time points, with an increase at 30 days. In the medial temporal cortex, PSEN2 was transiently upregulated at 2 days, with no further significant changes. Overall, these findings demonstrate a temporal shift toward amyloidogenic APP processing in the early phase following ischemia (Table 1).

Subsequent investigations, including those by Babusikova et al. (2021) [22], have shown that acute global ischemia leads to increased expression of APP and BACE1 at both the mRNA and protein levels, accompanied by a reduction in Aβ-degrading enzymes such as neprilysin (NEP), endothelin-converting enzyme-1 (ECE-1), and insulin-degrading enzyme (IDE). Accumulation of amyloid contributes to the activation of intracellular pathways in post-ischemic neurons, astrocytes, and microglia, thereby exacerbating neuronal and glial injury and promoting cell death following ischemia [22]. Moreover, increased permeability of the blood–brain barrier facilitates the infiltration of inflammatory cytokines and soluble Aβ into the post-ischemic brain [18,72,76]. The impact of cerebral ischemia and reperfusion on tau phosphorylation has also been extensively examined in experimental stroke models [77,78,79,80,81,82]. Elevated levels of tau protein have been detected in human plasma after ischemic brain injury and are associated with the progression of post-ischemic neurodegeneration [72]. Fujii et al. (2017) [81] demonstrated that both 3-repeat and 4-repeat tau isoforms undergo hyperphosphorylation during cerebral ischemia/reperfusion (I/R), resembling the pathological changes observed in Alzheimer’s disease. In addition, cerebral I/R induces the formation of neurotoxic tau species, including a 60 kDa Asp421-truncated form, followed by the generation of 17 kDa 3-repeat tau fragments, 25 kDa 4-repeat tau fragments, and multiple hyperphosphorylated tau variants [81].

## 4. The Role of S1P in AD

Alzheimer’s disease is a progressive and irreversible neurodegenerative disorder characterized by the extracellular accumulation of Aβ peptides, the formation of neurofibrillary tangles composed of hyperphosphorylated tau, and widespread neuronal loss [83]. Increasing clinical evidence indicates that inflammatory and immune-mediated processes play a significant role in AD pathogenesis. Accumulated Aβ has been shown to activate immune-related transcription factors, including NF-κB, and to promote the release of pro-inflammatory mediators such as TNF-α, COX-2, and iNOS via interaction with receptors like TLR2 and TLR4 (Toll-like receptors) [84]. Inflammatory signaling within neurons can directly modulate the activity of protein kinases and phosphatases, including ERK and protein phosphatase 2A (PP2A), thereby influencing tau phosphorylation and microtubule stability. Both the classical amyloid cascade hypothesis and inflammation-driven mechanisms are considered central to AD development. Moreover, alterations in sphingolipid metabolism and their bioactive derivatives in the brain, together with their impact on neuronal homeostasis and immune responses, may provide further insight into the underlying mechanisms of AD. Evidence from post-mortem human brain studies and experimental models suggests that dysregulation of S1P metabolism is a key feature associated with AD pathology [85,86,87]. The study by Couttas et al. (2014) [86] reported a decrease in S1P levels with advancing Braak stage in tissue samples from the CA1 region of the hippocampus and from the gray and white matter of the inferior temporal gyrus. Sphk2 activity decreased in the hippocampus and temporal gray matter, while the Sphk1 isoform declined only in the hippocampus [86]. The data of Lei et al. (2019) have confirmed reductions in Sphk2 activity and S1P loss during the early stage of AD, as well as an inverse correlation between female age and hippocampal S1P levels [88]. Moreover, Jęśko et al. (2019) demonstrated lower gene expression of Sphk2 in the brain cortex and hippocampus of 12-month-old AD transgenic mice carrying the V717I (‘London’) mutation, as well as downregulation of the genes encoding Sphk1 and Sphk2 in the post-mortem human AD hippocampus [89]. It was also documented that FTY720 increased Sphk2 mRNA levels in both brain regions affected in AD transgenic mice. Conversely, Takasugi et al. (2011) reported increased relative Sphk2 activity, normalized to Sphk2 protein levels, in the cerebral cortices of AD patients compared with non-demented subjects [90]. It can also explain the dual nature of Sphk2, which, depending on subcellular localization, may exert opposite functions within the cell, and the translocation of Sphk2 to the nucleus plays a crucial role in the switch between them [86]. Furthermore, S1P synthesized by Sphk2 was reported to increase the *in vitro*/*in vivo* proteolytic activity of BACE1, leading to the conclusion that Sphk2/S1P are required controllers of APP processing into Aβ [88,90]. It was demonstrated that Sphk2 preferentially localizes to the nucleus in the AD brain, while a reduced cytosolic Sphk2 pool correlated with the density of Aβ deposits [91]. Importantly, reduced Aβ load in Sphk2 null mice was accompanied by neurodegeneration, as evidenced by oligodendrocyte loss, reduced hippocampal volume, and hypomyelination, as well as worsened memory capacity. All these findings led to the conclusion that S1P loss is a strong inducer of neurodegeneration, surpassing the role of Aβ burden and pointing to the neuroprotective role of Sphk2/S1P signaling in AD pathology [88].

**Figure 4:**
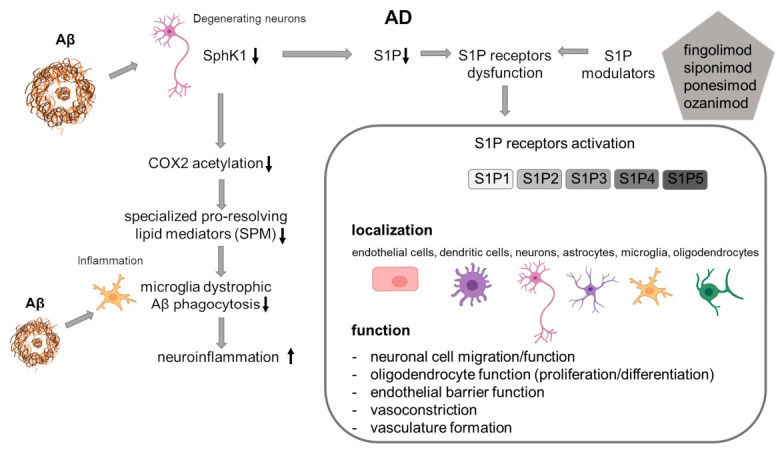
Sphingosine kinase 1 (Sphk1) and sphingosine-1-phosphate (S1P) receptor signaling pathway in Alzheimer’s disease (AD). During AD, amyloid beta (Aβ) deposition influences neuronal functioning and, in consequence, results in degenerated neurons that are deficient in Sphk1. Neurons induce microglial phagocytic dysfunction due to disrupted neuronal Sphk1 and COX2 signaling, reduced COX2 acetylation, and production of SPMs. Affected microglial phagocytic activity causes reduced Aβ phagocytosis and elevated neuroinflammation. Furthermore, during AD, sphingosine-1-phosphate levels are decreased, and in consequence, the signaling via S1P was disrupted. The role of S1P receptor modulators is widely studied in AD. The figure summarizes the pleiotropic mechanisms of S1P receptor activation by its modulators. Arrows terminating with ↑ = increased, arrows terminating with ↓ = decreased.

Similarly, Ceccom et al. (2014) [85] observed reduced immunoreactivity for Sphk1 and S1PR1, accompanied by increased S1P lyase activity, in the frontal and entorhinal cortices of human AD brains. Notably, reduced Sphk1 expression and elevated S1P lyase levels correlated with Aβ deposition in the entorhinal cortex of AD patients [85]. In an animal model of AD, S1P content decreased, whereas ceramide content increased after Sphk1-siRNA treatment of APP/PS1 mice. Furthermore, silencing of Sphk1 potentiated Aβ deposition and impairs learning and memory abilities [92]. The research by Jung et al. (2023) [93] showed substantial reductions in Sphk1 levels, increased levels of S1P lyase (S1PL) and S1PR1 in the 8- and 14-month-old 5xFAD mice when compared to non-transgenic wild type (WT) counterparts. Additionally, activation of the downstream signaling pathway involving Akt/mTOR/Tau was observed in aging 5xFAD mice [93]. It has been shown that S1PR1 activity is increased in the inner layers of the frontal cortex and in the underlying cortical white matter at early stages of AD. In contrast, S1PR1 receptor activity is decreased in the hippocampus during advanced stages of AD. Importantly, significant correlations between S1PR1 receptor activity and Braak stages suggested that S1PR1 receptor dysfunction is associated with disease progression [94]. Furthermore, decreases in S1P levels in the cerebrospinal fluid and blood of patients with early AD, as well as their correlation with AD pathology progression and severity, have been observed [95]. All this suggests that Sphk1/S1P decline may exacerbate Aβ-induced pathology, confirming the therapeutic potential of modulating the S1P/ceramide ratio in favor of S1P. Such a key role of Aβ in regulating Sphks and other enzymes involved in sphingolipid metabolism has been confirmed in both *in vitro* and *in vivo* studies, including models exposed to extracellular Aβ oligomer toxicity [96,97] and those with genetically induced APP overexpression [89,98,99]. Importantly, these studies demonstrated that changes in Sphk1 expression and activity may vary with exposure duration and Aβ concentration and do not always result in downregulation. For example, *in vitro* studies by Cieślik et al. (2015) showed that Aβ_1_–_42_ oligomers enhanced Sphk1 expression and activity in PC12 cells after 24 hours of incubation, together with elevated expression of sirtuin 4, superoxide dismutase 2, and catalase, whereas prolonged exposure (96 hours) led to downregulation of Sphk1 [97]. Differences in Sphk1 levels between shorter and prolonged incubation periods likely reflect the severity of oxidative stress induced by Aβ. However, an initial increase in Sphk1 activity is not always observed after Aβ exposure. Gomez-Brouchet et al. (2007) [96] reported fluctuating but consistently reduced Sphk1 activity in SH-SY5Y cells at several time points up to 24 hours. This decrease correlated with an increased ceramide/S1P ratio and was accompanied by progressive cell death. The mechanism was redox-sensitive, as N-acetylcysteine fully prevented Sphk1 downregulation and largely inhibited Aβ-induced cytotoxicity. Moreover, Sphk1 overexpression attenuated Aβ toxicity, whereas Sphk1 knockdown mimicked Aβ-induced cell death, highlighting Sphk1’s critical protective role [96]. In accordance with the effects of exogenously added Aβ described above, in PC12 cells producing endogenous Aβ (from either wild-type or Swedish-mutant APP), the expression and activity of Sphk1/2, as well as levels of the S1P1 receptor, were significantly reduced [98]. Consistent with these *in vitro* findings, *in vivo* studies in transgenic AD models further emphasize the role of Sphks in disease progression. Jęśko et al. (2019) [89] demonstrated that the AβPP (V717I) transgene led, with age, to reduced mRNA expression of S1PRs, Sphk2, ceramide kinase (CERK), and the anti-apoptotic Bcl2 in the cerebral cortex and hippocampus of 12-month-old mice, indicating a pro-apoptotic shift. These alterations largely mirrored changes observed in the human sporadic AD hippocampus, including reduced Sphk1, Sphk2, CERK, S1PR1, and Bcl2 [89]. The importance of changes in Sphks during Aβ-induced cell death is well established, although oxidative stress and neuroinflammation, which coexist, also play major roles in protein-mediated toxicity. Beyond its role in S1P biosynthesis, Sphk1 modulates COX-2 activity, thereby regulating the balance between prostanoid production and specialized pro-resolving mediators (SPMs). This Sphk1–COX-2–SPM axis is profoundly dysregulated in Alzheimer’s disease in both animal models and human studies. Notably, AD pathology is associated with decreased Sphk1 expression and a functional shift in COX-2 toward prostanoid generation [100,101,102] (see Figure 4 for a summary).

## 5. Beneficial Effects of S1P Receptor Modulators in Aβ Toxicity and Ischemia/Reperfusion Injury

The role of S1PR modulators is being extensively studied in both AD and ischemia/hypoxia models. Most research to date has focused on FTY720’s neuroprotective properties, given its long clinical use. Recently, there has been a clear shift toward evaluating a new generation of S1P modulators, such as siponimod and ponesimod. Studies are increasingly investigating their potential protective effects in AD and ischemia models. The tables below summarize the pleiotropic mechanisms of S1PR modulators, emphasizing FTY720 while also highlighting new modulators. These mechanisms include actions on immune cells and provide direct neuroprotection to neural and glial cells. This positions S1PR modulators as promising neuroprotective agents. S1PR modulators show significant anti-inflammatory and neuroprotective effects in models of AD (Table 2) and cerebral ischemia (Table 3). The protection is not always observed, especially in ischemia models [103,104,105]. While specific pathways and outcomes differ by pathological context, S1PR modulators share several mechanisms. These include anti-inflammatory actions, prevention of synaptic dysfunction, reduction in pathological protein aggregation, and modulation of apoptosis- and neuroprotection-related signaling (Table 4).

However, interpretation of receptor subtype-specific effects remains limited, as most studies have been conducted using compounds that target multiple S1P receptor subtypes simultaneously, and treatment outcomes may vary depending on the experimental model, disease stage, and cellular context.

Available evidence indicates that modulation of S1P receptors has broad therapeutic potential in AD. Reported benefits include reduced amyloid burden, decreased neuroinflammation, preserved synaptic integrity, and improved cognitive performance in various genetic and pharmacological AD models. These effects are often associated with reduced microglial and astroglial activation and modulation of pathways related to Aβ clearance, tau pathology, and sphingolipid homeostasis. However, mechanistic understanding remains limited. Most studies have used FTY720, a non-selective S1P receptor modulator, which complicates attribution to specific receptor subtypes. Limited studies with receptor-selective compounds, such as ozanimod and ponesimod, suggest that S1PR1 signaling is important [106,113]. Further research is needed to clarify receptor-specific roles in AD pathology.

Summarized studies show that S1P receptor modulation is primarily neuroprotective in acute ischemic, hypoxic, and chronic cerebral hypoperfusion models, as was observed in AD models. The beneficial effects include reduced infarct volume, preserved BBB integrity, reduced neuroinflammation, suppressed apoptotic signaling, and improved neurological or cognitive outcomes, recapitulating mechanisms observed in AD studies using S1P modulators. However, clear conclusions about the role of individual receptor subtypes in ischemic injury remain unclear. Results with the non-selective modulator FTY720 are inconsistent, showing strong neuroprotection [120,121,124,125,126], no effect [103], or even harm to BBB integrity [104]. Similarly, Siponimod reduced lymphocyte infiltration but did not improve infarct size or function [105]. Overall, the effects of S1P signaling in cerebral ischemia depend on receptor subtype, disease model, and BBB status.

The data in Table 4 show that S1P receptor modulators affect several interconnected molecular and cellular pathways involved in both AD and ischemia or hypoxia-related pathology. The most consistently affected processes are inflammatory signaling (including IL-1β, IL-6, and TNF-α), microglial and astrocyte activation, and BBB integrity. In both disease models, modulation of S1P signaling is often associated with suppression of the NF-κB, MAPK, and TLR4 pathways, reduced glial activation, and improved vascular stability. Several studies also report activation of pro-survival pathways, especially PI3K/Akt/mTOR, as well as changes in oxidative stress markers and mitochondrial function. In AD, additional effects include changes in BDNF regulation and sphingolipid metabolic enzymes, suggesting broader roles in neurotrophic and lipid homeostasis. Overall, S1P modulation provides anti-inflammatory and neuroprotective effects across models, but the diversity of molecular targets and outcomes indicates a complex, context-dependent mechanism.

Current clinical evidence suggests that fingolimod may be a promising treatment for acute ischemic stroke. Studies have reported better neurological recovery, less infarct progression, and fewer cases of hemorrhagic transformation in patients who received fingolimod in addition to standard treatment. While these results show the potential of S1P receptor modulation in stroke, the data so far come from relatively small patient groups. Larger randomized controlled trials are needed to confirm these findings, identify which patients benefit most, and define the role of fingolimod in stroke treatment [59].

Recent progress in the clinical development of S1P receptor modulators has enhanced the therapeutic prospects of targeting S1P signaling in neurodegenerative disorders. Although no S1P-targeting therapy has been approved for Alzheimer’s disease, several modulators are currently undergoing clinical evaluation. Siponimod (NCT06639282) and ozanimod (NCT06803823, NCT06881836) have reached Phase II clinical trials in patients with Alzheimer’s disease. Investigation of these compounds is based on their ability to modulate neuroinflammation, maintain blood–brain barrier integrity, and regulate microglial activation [130].

## 6. Conclusions

S1P-associated signaling pathways play a multifaceted and potentially important role in the pathogenesis and treatment of cerebral ischemia and AD. The dual activity of Sphk1 and Sphk2 kinases underscores the complex, context-dependent nature of S1P signaling. In addition, S1PRs exhibit a broad spectrum of effects depending on receptor subtype and cellular state, with pathological conditions often acting as triggers for changes in S1PR expression. While activation of S1PR1 and S1PR5 generally supports BBB integrity and neuronal survival, S1PR2, S1PR3, and S1PR4 tend to promote vascular permeability, neuroinflammation, and ischemic damage.

There is a significant overlap in pathological mechanisms between ischemic stroke and AD, including BBB disruption, chronic inflammation, amyloid pathology, and tau hyperphosphorylation. Modulation of the S1P/Sphks/S1PRs axis represents a promising shared therapeutic strategy. Notably, S1P receptor modulators such as FTY720, siponimod, ponesimod, and ozanimod, which are currently approved for the treatment of multiple sclerosis, have also demonstrated neuroprotective effects in experimental models of both cerebral ischemia and AD. Pharmacological targeting of S1P signaling not only has therapeutic promise for demyelinating diseases but also serves as a potential intervention point in the context of neurodegeneration and stroke. Continued investigation into selective S1PR modulators may pave the way for new treatment strategies that address the complex interplay between vascular dysfunction and neuronal loss.

## Figures and Tables

**Table 1:** Regulation of Enzyme Gene Expression Concerning APP Metabolism Following Cerebral Ischemia.

Molecular Targets	Expression and Activity Changes in Ischemia/Hypoxia Models
ADAM10, ADAM17	ADAM10 mRNA ↓ in hippocampal CA3 at 2, 7 and 30 days post-ischemia [73] ADAM17 mRNA levels and activity ↓ in hippocampus in chronic cerebral hypoperfusion [74]
beta-secretase 1 (BACE1)	mRNA ↑ in CA1 at 2 and 7 days post-ischemia, ↓ after 30 days mRNA ↓ in CA3 at 2 and 7 days post-ischemia, ↑ at 30 days mRNA ↑ in the medial temporal cortex at 2 days post-ischemia, ↓ at 7 and 30 days [73,75]
presenilin 1 (PSEN1)	mRNA ↑ in CA1 and CA3 at 2 and 7 days post-ischemia–reperfusion, ↓ at 30 days mRNA ↔ in medial temporal cortex at 2, 7 and 30 days post-ischemia–reperfusion [73,75]
presenilin 2 (PSEN2)	mRNA ↑ in CA1 at 2 and 7 days after ischemia–reperfusion, ↓ at 30 days mRNA ↓ in CA3 at 2 and 7 days after ischemia–reperfusion, ↑ at 30 days mRNA ↑ in the medial temporal cortex at 2 days after ischemia–reperfusion, ↔ at 7 and 30 days [73,75]

↑ = increased, ↓ = decreased, ↔ = unchanged/no effect.

**Table 2:** The Role of S1PR Modulators in the AD Model Since 2020.

S1P Modulator–S1P Receptor Preferential Selectivity	Treatment Regimen and Disease Model	The Effect of S1P Modulators in the AD Model	Ref.
Ozanimod (RPC1063)–S1PR1, S1PR5	1 mg/kg/day for 45 days, oral administration via drinking water, 16–17-month-old APP/PS1 mice (10 months after onset of cognitive deficits)	Ozanimod mitigated reduced spine density, decreased Aβ burden, and reduced microgliosis and astrogliosis in the hippocampus and neocortex.	[106]
FTY720–S1PR1, S1PR3, S1PR4, S1PR5	1 mg/kg daily for 2 months, oral, 8-month-old APP/PS1 mice	FTY720 improved cognition and reduced Aβ accumulation, tau hyperphosphorylation, and neurodegeneration via decreased lymphocyte infiltration and neuroinflammation, as well as downregulation of AD-related proteins.	[107]
S1P analogs cP1P and P1P	0.1, 1 mg/kg, i.p., administered on alternate days for 4 weeks, starting 4 weeks after Aβ injections, in C57BL/6J mice	cP1P and P1P decreased Aβ deposition and hyperphosphorylated Tau, attenuated gliosis and neuroinflammatory signaling (GFAP, Iba-1, p-NF-κB, TNF-α, IL-1β), influenced S1PR1-Akt/mTOR pathways, limited mitochondrial stress reducing p-JNK, Caspase-3, PARP-1 expression, modulated synaptic markers (PSD-95, SNAP-25, Syntaxin), enhanced behavioral performance in Aβ-treated mice, and supported neuronal survival *in vitro*.	[108]
FTY720–S1PR1, S1PR3, S1PR4, S1PR5	Carboxymethylcellulose-encapsulated FTY720, siRNA and ZnO hybrid nanocomposite administered to immortalized microglial cells (IMG) treated with Aβ oligomers (Aβo)	FTY720 treatment normalized microglial priming, reduced levels of pro-inflammatory mediators, enhanced BDNF secretion, and improved Aβ phagocytosis.	[109]
Phosphorylated FTY720 (pFTY720)–S1PR1, S1PR3, S1PR4, S1PR5; Ponesimod–S1PR1; CYM5541–S1PR3; CYM50308–S1PR4; A971432–S1PR5; Siponimod–S1PR1, S1PR5.	S1PR modulators administered with 1 μM Aβo for 24 h to hippocampal neuronal (HT22) and microglial (BV2) cell lines	Exacerbation of Aβo-induced proinflammatory effects in microglial cells was indicated by further increases in IL-1β levels following treatment with siponimod, ponesimod, and S1PR4/5 modulators, as well as by the upregulation of BAX expression (ponesimod, S1PR3/4 modulators) or the BAX/BCL2 ratio (ponesimod, pFTY720, and the S1PR5 modulator) during co-incubation with Aβo. In HT22 cells, S1PR4 and S1PR5 agonists restored BCL2 mRNA levels reduced by Aβo, while pFTY720 suppressed Aβo-induced IL-6 upregulation. Notably, co-incubation of siponimod with Aβo increased IL-1β levels compared to Aβo treatment alone.	[110]
FTY720–S1PR1, S1PR3, S1PR4, S1PR5	0.03 mg/kg/day in drinking water for 7 months, 5xFAD mice	FTY720 reversed the reduced levels of Sphk1 and Sphk2 kinases and decreased the elevated level of S1P lyase, as well as the elevated ratios of p-Akt (Ser473)/Akt, p-S6 (Ser240/244)/S6, and p-Tau (Ser202)/Tau, along with S1PR1 expression, in 5xFAD mice.	[93]
FTY720–S1PR1, S1PR3, S1PR4, S1PR5	1 mg/kg, i.p., once daily for 2 weeks; obese/prediabetic mice (indirect AD model based on obesity-associated dementia risk)	FTY720 yielded anxiolytic effects and restored cognition-related behaviors, reduced pro-inflammatory cytokines in the cortex and hippocampus, and decreased BACE1, PSEN2, and GSK3β levels in the cortex.	[111]
FTY720–S1PR1, S1PR3, S1PR4, S1PR5	1 mg/kg/day in drinking water for 50 days in APPPS1-21 mice (APP Swedish + PS1 L166L); treatment initiated at two time points: 50 days (early, pre/very early plaque deposition) and 125 days (late, advanced plaque deposition), male and female mice	FTY720 administration in the late paradigm reduced Aβ burden in male APPtg mice, decreased microglial activation and IL-1β levels, and had no significant effects in females treated at the late stage or in both sexes of mice treated earlier, before Aβ accumulation.	[112]
Ponesimod–S1PR1	10 or 100 nM ponesimod and 1 μM Aβ_42_ for 24 h on primary glial cultures; 30 mg/kg, oral gavage, once daily for 4 weeks in 5XFAD mice	Ponesimod disrupted the TLR4–S1PR1 complex and suppressed STAT1, pERK, p38, and JNK signaling while activating STAT6. It enhanced microglial phagocytosis and Aβ clearance. *In vivo*, it reduced TNF-α, CXCL10, Iba-1^+^ microglia, GFAP^+^ astrocytes, and amyloid plaques, increased IL-33, and improved spatial memory.	[113]
FTY720–S1PR1, S1PR3, S1PR4, S1PR5	1 mg/kg, i.p.; 3- and 12-month-old TgAD (AβPP V717I) mice, and 3-month-old mice in a streptozotocin-induced sporadic AD (SAD) model	The drug normalized mGluR3 transcription in the TgAD model and downregulated VGLUT1, AMPAR2, and mGluR3 expression in the SAD model.	[114]
FTY720–S1PR1, S1PR3, S1PR4, S1PR5	1 mg/kg/day in drinking water from 6 to 12 months of age, starting after the emergence of behavioral symptoms; 3xTg-AD mice	FTY720 improved spatial working memory, reduced brain inflammation (fewer Iba-1^+^ cells in CA1/CA3, less ramified microglia, a favorable cytokine profile, decreased circulating CD3^+^ T cells, and those infiltrating the cortex), and lowered phosphorylated tau and APP levels in the hippocampus and cortex.	[115]
FTY720–S1PR1, S1PR3, S1PR4, S1PR5	5 mg/kg, i.p., daily for 7 days; APP/PSEN1 transgenic mice (amyloid precursor protein and presenilin)	FTY720 significantly ameliorated psychosis-related behavioral changes and broadly upregulated synaptic protein levels in cortical homogenates. Notably, a distinct cluster of mitochondria-associated proteins—most prominently affected by the treatment—correlated with behavioral improvements.	[116]
FTY720–S1PR1, S1PR3, S1PR4, S1PR5	1 mg/kg, i.p., daily for 2 weeks; 3- or 12-month-old female FVB-Tg (Thy1; APP LD2/B6) mice overexpressing human amyloid precursor protein (APP) with the V717I (‘London’) mutation	FTY720 normalized the APP-induced reduction in the expression of Complexin 1 (Cplx1), Syntaxin 1A (Stx1a), Synaptosomal-associated protein 25 (Snap25), and Neurexin 1 (Nrxn1) in the hippocampus of 12-month-old mice, which exhibited high vulnerability to early neurotoxic damage. However, in the cortex, restoration was observed only for Vesicle-associated membrane protein 1 (Vamp1). No effect of FTY720 was detected in the brains of 3-month-old mice.	[99]
FTY720–S1PR1, S1PR3, S1PR4, S1PR5	Identification of AD-associated FTY720 targets (F-ADGs) through intersection analysis of frontal cortex expression profiles (423 AD, 266 control); Analysis of miRNA data from the frontal cortex of AD patients to identify severity-related F-ADG-miRNAs	A total of 188 F-ADGs, mainly associated with synaptic function, inflammation, and reactions to oxygen-containing compounds, were detected in the frontal cortices of AD patients. miRNAs were likely involved, with key targets identified as S1PR1, Aldehyde Dehydrogenase 1 Family Member L1 (ALDH1L1), Formyl Peptide Receptor 1 (FPR1), and Gamma-Aminobutyric Acid Type B Receptor Subunit 2 (GABBR2).	[117]
FTY720–S1PR1, S1PR3, S1PR4, S1PR5	1 mg/kg, i.p., daily for 2 weeks; female FVB-Tg (Thy1; APP LD2/B6) mice overexpressing human APP with the V717I mutation, aged 3, 6, or 12 months	FTY720 inhibited the APP-induced increase in hippocampal ceramide synthase 2 (CERS2) in 3-month-old mice and mitigated APP-associated reductions in sphingomyelin synthase 1 (SGMS1) and 2 (SGMS2) at 12 and 6 months, respectively. It also downregulated alkaline ceramidase 2 (ACER2) mRNA in 6-month-old healthy controls, as well as alkaline ceramidase 3 (ACER3) and ceramide synthase 4 (CERS4), both of which remained unchanged in 12-month-old untreated APP mice. Furthermore, FTY720 upregulated acid ceramidase (ASAH1) and sphingomyelin phosphodiesterase 2 (SMPD2), which were unchanged in both 6- and 12-month-old untreated APP mice.	[118]
FTY720–S1PR1, S1PR3, S1PR4, S1PR5	1 mg/kg, i.p., every other day for 4–6 weeks, starting after symptom onset (at 22–24 weeks of age); APP/PS1 mice with the APP ‘Swedish mutation’ (KM670/671NL) and PS1 Leu166Pro mutation	FTY720 rescued synaptic pathology thought to contribute to memory deficits in AD mice when given after symptom onset in the APP/PS1 model. It restored spine density and long-term potentiation (LTP), both of which were impaired in hippocampal CA1 pyramidal neurons, and improved spatial memory. Additionally, moderate Aβ reduction and a marked decrease in neuroinflammation were observed in the hippocampus and neocortex, as indicated by fewer Iba-1 and GFAP-positive glial cells.	[119]

**Table 3:** The Role of S1P Receptor Modulators in Cerebral Ischemia/Hypoxia Models Since 2020.

S1P Modulator–S1P Receptor Preferential Selectivity	Treatment Regimen and Disease Model	The Effect of S1P Modulators in the Cerebral Ischemia/Hypoxia Model	Ref.
A-971432–S1PR5 agonist	0.1 mg/kg, i.p., post-middle cerebral artery occlusion (MCAO), followed by once-daily injections for 3 consecutive days in a mouse MCAO (ischemia/reperfusion) model	A-971432 reduced infarct volume and neuronal injury in the ischemic cortex and hippocampus, suppressed apoptosis and inflammation, by activating the PI3K/Akt/mTOR and inhibiting the p38/ERK/JNK pathway; S1PR5-dependent effects were confirmed by receptor knockdown, which exacerbated injury.	[71]
NXC736–S1PR4 functional antagonist	3 mg/kg, p.o., 1 h post-occlusion in a mouse permanent MCAO (pMCAO) model	NXC736 improved functional outcome, reduced infarct size and apoptosis, and limited post-ischemic BBB disruption and edema; it also attenuated microglial activation, oxidative stress, and pro-inflammatory signaling, linked to suppression of NF-κB and MAPK pathways (ERK1/2, JNK, p38).	[70]
FTY720–S1PR1, S1PR3, S1PR4, S1PR5	0.5 (low), 1.0 (medium), and 2.0 (high) mg/kg, i.p., on days 0 and 1 after transient MCAO (tMCAO)	The drug dose-dependently reduced infarct volume and brain edema and supported neurological recovery in rats that underwent MCAO/R. Moreover, it decreased serum proinflammatory cytokines (IL-1β, IL-6, TNF-α) and hippocampal high-mobility group box 1 (HMGB1)/TLR4/nuclear factor kappa-light-chain-enhancer of activated B cells (NF-κB) expression.	[120]
FTY720–S1PR1, S1PR3, S1PR4, S1PR5	1 mg/kg, i.p., immediately post-reperfusion, followed by once-daily injections for up to 7 days in a rat MCAO model	FTY720 provided protection against neurovascular unit injury, improved neurological deficits, and promoted functional recovery after ischemic stroke. It reduced infarct size, BBB leakage, and edema, and reversed the ischemia-induced reduction in tight junction proteins in microvessels. Additionally, FTY720 lowered S1PR1 expression in microvessels and decreased glial activation, IL-17A protein levels, and its colocalization with Iba-1 and GFAP.	[121]
FTY720–S1PR1, S1PR3, S1PR4, S1PR5	0.3 mg/kg, i.p., 45 min post-hypoxia onset, daily for 12 days until postnatal day 21 (P21, lactation period) in a rat pup hypoxia-induced neonatal seizure (HINS) model	FTY720 administration during the lactation period mitigated HINS-induced cognitive impairments by reducing oxidative stress, as indicated by lower hippocampal malondialdehyde (MDA) levels, in both male and female juvenile rats. It did not reverse the decline in BDNF, the increase in nitric oxide (NO), or the reduction in interleukin-4 (IL-4). However, FTY720 decreased hippocampal TNF-α expression only in male hypoxic animals and interacted with sex in modulating both TNF-α and BDNF levels.	[122]
Ozanimod–S1PR1, S1PR5	0.5 nM; preincubation before Hypoxia plus Glucose Deprivation (HGD) in Human Brain Microvascular Endothelial Cell (HBMEC) culture	Ozanimod attenuated HGD-induced loss of HBMEC integrity by reducing MMP-9 activity and preserving claudin-5 and PECAM-1 levels via S1PR1 activation.	[123]
FTY720–S1PR1, S1PR3, S1PR4, S1PR5	Pre-treatment: 0.5 (low) or 2 mg/kg (high), i.p., once daily for 7 days before tMCAO/R Post-treatment: same regimen initiated immediately post-reperfusion	FTY720 (administered both before and after operation) reduced neurological scores and neuronal damage, as indicated by increased Nissl bodies in the cerebral cortex compared to the operated groups. Additionally, post-treatment with FTY720 improved memory impairment induced by tMCAO/R. Both treatment regimens suppressed the release of inflammatory cytokines (IL-1β, IL-6, TNF-α) in the cerebral cortex and hippocampus and attenuated p38MAPK and NF-κB phosphorylation in the hippocampus, thereby downregulating inflammatory signaling in the brain.	[124]
FTY720–S1PR1, S1PR3, S1PR4, S1PR5	1 mg/kg, i.p., 30 or 60 min post-occlusion and 24 h later in permanent distal and transient MCAO mouse models, respectively; active/inactive circadian phases	FTY720 lowered peripheral lymphocyte counts in naïve mice but did not significantly influence infarct size, brain edema, hemorrhagic transformation, or motor and cognitive outcomes assessed 2–3 days after either transient or permanent focal cerebral ischemia, regardless of whether treatment occurred during the active or inactive circadian phase.	[103]
FTY720–S1PR1, S1PR3, S1PR4, S1PR5	1 mg/kg, i.p., for 7 weeks starting on post-operative day 8 in a rat two-vessel occlusion (2VO) model of chronic cerebral hypoperfusion (CCH)	FTY720 prevented spatial memory loss in 2VO rats, which was linked to reduced neuroinflammation, including lower levels of pro-inflammatory cytokines (TNF-α, IL-1β, IL-6) and fewer Iba-1-positive cells in the hippocampus. It also improved mitochondrial dysfunction, as indicated by decreased MDA, increased ATP, and enhanced ATP synthase activity in the hippocampus. No effect was observed on CCH-induced reduction in hippocampal SIRT-3 activity, suggesting an SIRT3-independent pathway.	[125]
FTY720–S1PR1, S1PR3, S1PR4, S1PR5	1 mg/kg, i.p., immediately post-reperfusion after 1 h tMCAO in adult male rats with streptozotocin-induced hyperglycemia	FTY720 negatively affected BBB integrity (exacerbating brain edema and reducing ZO-1, Occludin, and S1PR1 protein levels by MCAO), which outweighed its neuroprotective and anti-inflammatory effects (decreased inflammatory cell infiltration, reduced TNF-α mRNA, increased Bcl-2/Bax ratio), leading to no improvement in endpoint outcomes of diabetic mice 24 h post-tMCAO.	[104]
FTY720 and FTY720-P–S1PR1, S1PR3, S1PR4, S1PR5	0.5 (low) or 1.5 mg/kg (high), i.p., immediately pre-reperfusion in a rat tMCAO model; FTY720 or FTY720-P (100 nM) before reperfusion in an OGD/reperfusion model using BBMVECs	FTY720 reduced infarct size and mortality in a dose-dependent manner, with functional recovery improvements independent of dosage. It inhibited the progression of inflammation during the subacute phase in the peri-infarct areas by reducing Iba-1-positive activated microglia and macrophages, suppressing apoptosis, and enhancing BBB integrity. *In vitro*, FTY720-P preserved the intracellular redistribution of tight (ZO-1) and adherens (VE-cadherin) junction markers without affecting their mRNA expression, in a manner dependent on the S1PR1 receptor, and also tended to protect the BBB.	[126]
Siponimod–S1PR1, S1PR5	3 mg/kg, i.p., for 6 consecutive days in a mouse tMCAO model	Siponimod induced significant lymphopenia in peripheral blood and reduced T-cell infiltration in the CNS on day 7 after tMCAO; however, it did not affect lesion size or behavioral functional outcomes.	[105]

**Table 4:** The Role of S1PR Modulators in Signaling Pathways Associated with the Pathology of AD and Ischemia.

Molecular Target	AD Model	Ischemia/Hypoxia Model
**Inflammation Signaling Pathways**		
↑ Expression and Secretion of Pro-inflammatory Mediators in Microglial Cells	↑ Pro-inflammatory cytokine release (TNF-α, IL-1β, IL-12) induced by Aβo in IMG cells → ↓ Secretion after treatment with FTY720-loaded hybrid nanocomposite [109]. ↑ IL-1β (cortex) and IL-6/TNF-α (cortex and hippocampus) → ↓ after FTY720; IL-6 in both regions, IL-1β and TNF-α in cortex only [111]. ↑ IL-1β mRNA in microglial cell line after Aβo treatment → further ↑ with siponimod, ponesimod, and S1PR4/5 modulators (exacerbating proinflammatory effect) ↑ IL-6 mRNA expression in HT22 and BV2 cells after Aβo administration → significant ↓ with pFTY720 treatment in HT22 cells. ↓ mRNA IL18 in both BV2 and HT22 cells [110]. ↓ IL-1β in male brain tissue after FTY720 administration in APPtg mice, starting post-plaque deposition [112]. ↓ TNF-α and CXCL10 levels after Ponesimod treatment in 5xFAD mice [113].	↑ Proinflammatory cytokines (IL-1β, IL-6, TNF-α) in serum of MCAO/R rats → ↓ cytokine levels after FTY720 treatment [120]. ↑ astrocyte/microglia and IL-17A colocalization → FTY720 ↓ colocalization and ↓ IL-17A protein level [121]. ↑ TNF-α gene expression in hippocampus of juvenile male rats subjected to HINS → ↓ after FTY720 [122]. ↑ IL-1β, IL-6, and TNF-α mRNA and protein levels in the hippocampus and cerebral cortex of tMCAO/R rats → ↓ in FTY720-treated groups administered pre- and post-tMCAO operation [124]. ↑ IL-1β, IL-6, and TNF-α concentration in the hippocampus of 2VO rats → ↓ after FTY720 [125]. ↑ TNF-α mRNA in the brain 24 h after tMCAO → ↓ after acute (immediately after reperfusion) FTY720 treatment [104].
Iba-1^+^ Microglia and GFAP^+^ Astrocytes Count	↑ reactive microglial density, altered morphology, ↑ astrogliosis → FTY720 ↓ Iba-1^+^ cells, restores resting morphology, ↓ GFAP^+^ cell number [115,119]. ↑astrocyte/microglia activation →↓ ozanimod microgliosis and astrogliosis [106]. Ponesimod ↓ Iba-1^+^ microglia and GFAP^+^ astrocytes in 5xFAD mice [113]. cP1P and P1P (S1P analogs) ↓ gliosis/neuroinflammatory signaling (GFAP, Iba-1, p-NF-κB, TNF-α, IL-1β) [108]	↑ astrocyte/microglia activation → FTY720 ↓ Iba-1^+^ and GFAP^+^ [121]. Iba-1^+^ activated microglia and macrophages ↓ dose-dependently by FTY720 in peri-infarct areas in tMCAO rat model [126].
Anti-inflammatory Markers	↑ IL-33 levels and Stat6 by ponesimod in 5xFAD mice [113].	↓ IL-4 gene expression in hippocampus of juvenile rats previously subjected to hypoxia → ↔ after FTY720 [122].
p38 MAPK, Stat1/Stat6, and TLR4 Signaling Pathway	↑ TLR4, S1PR1, and complex formation with Aβ42 → ↓ TLR4, S1PR1, complex formation, and Stat1 and p38 MAPK activation with ponesimod [113].	↑ protein levels of HMGB1, TLR4, and NF-κBp65 in the hippocampus of MCAO/R rats → ↓ after FTY720 [120]. ↑ phosphorylation of p38MAPK and NF-κB pathway-associated molecules in the hippocampus of tMCAO/R rats → ↓ after FTY720 administered before or after tMCAO/R [124] A-971432 (S1PR5 agonist)-mediated p38/ERK/JNK pathway inhibition [71] NXC736 (S1PR4 antagonist)-mediated inhibition of NF-κB and MAPKs (ERK1/2, JNK, and p38) signaling [70]
Blood–Brain Barrier Integrity	Disrupted microvessels, ↑ MMPs near Aβ plaques in 5XFAD mice → BBB breakdown [127]. ↓ Claudin-5, Occludin, ZO-1 in Aβ-laden capillaries in human cerebral amyloid angiopathy [128].	↓ tight junction proteins occludin and claudin-5 in the microvessels of ischemic stroke rats → ↑ protein expression after FTY720, ↑ neurovascular integrity, and stabilized BBB permeability [121]. ↑ MMP-9 activity and ↓ Claudin-5 and PECAM-1 protein levels → attenuation of these effects by ozanimod [123]. ↑ BBB permeability, brain edema after I/R injury, junctional proteins (ZO-1, VE-cadherin) translocated into cytoplasm → BBB integrity improved by FTY720, intracellular redistribution of tight junction proteins prevented by P-FTY720 post-I/R, ↔ their mRNA levels [126]. ↑ brain edema and disruption of BBB integrity, reduction in tight junction protein 1 (ZO-1), Occludin, and S1PR1 → FTY720 further aggravated brain edema and reduced the expression levels of tight junction proteins and S1PR1 [104]. NXC736 (S1PR4 antagonist) ↓ post-ischemic BBB disruption and edema [70].
Lymphocyte Infiltration in the CNS	↓ CD3^+^ lymphocytes in circulation and ↓ CD3^+^ lymphocyte infiltration in the cortex after FTY720 [115].	Siponimod ↓ lymphocyte count within the peripheral blood 7 days post-stroke and ↓ T-cells brain infiltration [105].
**Neuroprotection and Apoptosis**		
Akt/PI3K	S1P analogs P1P/cP1P-mediated activation of S1PR1 → PI3K/Akt/mTOR pro-survival signaling [108].	A-971432 (S1PR5 agonist) activates PI3K/Akt/mTOR in MCAO model [71].
BDNF	↓ Secretion following Aβo treatment in IMG cells → ↑ after FTY720 [109].	↓ BDNF → FTY720 did not prevent BDNF losses but it showed interaction with sex in hippocampal BDNF concentration [122].
Biomarkers of Oxidative/Nitrative Stress	↓ Mitochondrial membrane potential after Aβo exposure in HT22 and BV2 cells; ↔ with S1P modulators [110].	↑ MDA levels, ↓ ATP concentration/ATP synthase activity in the hippocampus of 2VO rats → FTY720 ↓ MDA, ↑ ATP level/ATP synthase activity [125]. ↑ MDA and NO in the hippocampus of both male and female juvenile hypoxic rats, ↑ thiol level in females → FTY720 administered during the lactation period ↓ MDA and thiol level, ↔ on the NO level [122].
**Sphingolipid Metabolism and Function**		
S1P Receptors and Sphingolipid Metabolism Enzymes	↑ Sphk1 and ↓ S1PR1 mRNA levels in the cortex and hippocampus of obese/prediabetic mouse brains [111]. ↓ mRNA levels of sphingomyelin synthases SGMS1 and SGMS2 in the neocortex and hippocampus of AD patients compared to age-matched controls. In V717I AβPP mice: ↑ enzymes of ceramide turnover on the salvage pathway (CERS2, CERS4, CERS6, and ACER3) at 3 months; ↑ CERS6 and ↓ SGMS2 at 6 months; ↓ SPHK2, CERK, and SGMS1 at 12 months. → After FTY720 treatment: ↓ CERS2 at 3 months; ↑ SGMS2 at 6 months; ↑ SPHK2, CERK, and SGMS1 at 12 months [118].	In a model of human induced pluripotent stem cell-derived cardiomyocytes (hiPSC-CMs) under I/R conditions: ↑ mRNA levels of CerS2/4 following IR, ↑ total ceramide, including long-chain (C16:0, C18:0, C18:1) and very long-chain (C22:0, C24:1) ceramide species following reperfusion compared to control or ischemic CMs → apoptosis, mitochondrial dysfunction → reversed by inhibition of ceramide formation with fumonisin B1 [129].

↑ = increased, ↓ = decreased, ↔ = unchanged/no effect.

## Data Availability

No new data were created or analyzed in this study. Data sharing is not applicable to this article.

## Abbreviations

The following abbreviations are used in this manuscript:

2VO	two-vessel occlusion
Aβ	amyloid beta
ACER3	alkaline ceramidase 3
AD	Alzheimer’s disease
ADAM10/17	A disintegrin and metalloproteinase domain-containing proteins 10 and 17
APP	amyloid precursor protein
BACE1	beta-site APP cleaving enzyme 1
BBB	blood–brain barrier
BDNF	brain-derived neurotrophic factor
CCH	chronic cerebral hypoperfusion
CERK	ceramide kinase
CERS	ceramide synthases
CNS	central nervous system
COX-2	cyclooxygenase-2
ERK1/2	extracellular signal-regulated kinases 1 and 2
FTY720	fingolimod
GPCRs	G protein-coupled receptors
ICAM	intercellular adhesion molecule
IL-1β	interleukin-1 beta
IL-4	interleukin-4
IL-6	interleukin-6
IL-12	interleukin-12
iNOS	inducible nitric oxide synthase
I/R	ischemia/reperfusion
JNK	c-Jun N-terminal kinase
LC3-II	microtubule -associated protein 1A/1B-light chain 3-II
MAPK	mitogen-activated protein kinase
MCAO	middle cerebral artery occlusion
MCAO/R	middle cerebral artery occlusion with reperfusion
mTOR	mechanistic target of rapamycin
MS	multiple sclerosis
NF-κB	nuclear factor kappa B
OGD	oxygen–glucose deprivation
pMCAO	permanent middle cerebral artery occlusion
PI3K/AKT	phosphoinositide 3-kinase/protein kinase B
PP2A	protein phosphatase 2A
PSEN1/2	presenilin 1 and 2
S1P	sphingosine-1-phosphate
S1PR1–S1PR5	sphingosine-1-phosphate receptors 1 to 5
SAD model	streptozotocin-induced sporadic Alzheimer’s disease model
SGMS1/2	sphingomyelin synthases 1 and 2
SGPL1	sphingosine-1-phosphate lyase 1
SGPPs	sphingosine-1-phosphate phosphatases
Siponimod (BAF312)	selective sphingosine-1-phosphate receptor modulator
Sphk1	sphingosine kinase 1
Sphk2	sphingosine kinase 2
tMCAO	transient middle cerebral artery occlusion
TLR	Toll-like receptor
TNF-α	tumor necrosis factor alpha
TREM2	triggering receptor expressed on myeloid cells 2
VCAM	vascular cell adhesion molecule
ZO-1	zonula occludens-1
i.p.	intraperitoneal
